# Scientific Trends in Clinical Research on Zirconia Dental Implants: A Bibliometric Review

**DOI:** 10.3390/ma13235534

**Published:** 2020-12-04

**Authors:** Felice Lorusso, Sammy Noumbissi, Inchingolo Francesco, Biagio Rapone, Ahmad G. A. Khater, Antonio Scarano

**Affiliations:** 1 Department of Medical, Oral and Biotechnological Sciences, University of Chieti-Pescara, Via dei Vestini, 31, 66100 Chieti, Italy; drlorussofelice@gmail.com (F.L.); sammy@iaoci.com (S.N.); 2 Zirconia Implant Research Group (Z.I.R.G), International Academy of Ceramic Implantology, Silver Spring, MD 20910, USA; 3 Department of Interdisciplinary Medicine, University of Bari Aldo Moro, 70121 Bari, Italy; f.inchingolo@icloud.com; 4 Department of Basic Medical Sciences, Neurosciences and Sense Organs, University of Bari Aldo Moro, 70121 Bari, Italy; biagiorapone79@gmail.com; 5 Faculty of Oral and Dental Medicine, Ahram Canadian University, 6th of October City, 8655 Giza, Egypt; ahmed.g.a.khater@gmail.com

**Keywords:** zirconia implant, bibliometrics, citations, scientometric

## Abstract

Background: The clinical use of zirconia implants has been shown to increase steadily due to their biological, aesthetic, and physical properties; therefore, this bibliometric study aimed to review the clinical research and co-authors in the field of zirconia dental implant rehabilitation. Methods: We searched Scopus and Web of Science databases using a comprehensive search strategy to 5 October 2020, and independently paired reviewers who screened studies, and collected data with inclusion criteria restricted to clinical research only (either prospective or retrospective). Data on article title, co-authors, number of citations received, journal details, publication year, country and institution involved, funding, study design, marginal bone loss, survival rate, failure, follow-up, and the author’s bibliometric data were collected and evaluated. Results: A total of 29 clinical studies were published between 2008 and 2020 as 41.4% were prospective cohort studies and 48.3% originated from Germany. Most of the included studies had been published in Clinical Oral Implant Research (*n* = 12), and the most productive institution was the Medical Center of University of Freiburg. The author with the largest number of clinical studies on zirconia implants was Kohal R.J. (*n* = 10), followed by Spies B.C. (*n* = 8). Conclusions: This study revealed that zirconia implants have been more prominent in the last ten years, which is a valuable option for oral rehabilitation with marginal bone loss and survival rate comparable to titanium dental implants.

## 1. Introduction

The clinical application of dental implant rehabilitation represents consolidated effectiveness in the literature due to long-term predictability and high-level satisfactory functioning and aesthetics [1,2,3,4]. Titanium alloys are the most widely used biomaterials for dental implant fixtures due to their physical, chemical, and thermal properties, which produce the osseointegrating ability of the fixture placed to replace the natural teeth [2,5,6,7,8,9,10,11,12].

Recently, the use of zirconia as an implant material has become more prevalent due to its high aesthetic characteristics, particularly in the rehabilitation of the compromised anterior jaw area, where there is fine soft-tissue biotype and the metal sensibility of the patients [13,14,15].

In fact, the literature reports that the titanium ion dissolution related to the implant corrosion could alter the natural oral microbiome and the homeostatic functional balance of the oral tissues [16,17,18,19,20].

On the contrary, it has been shown in vitro that the zirconia surface can lead to a significant decrease in periodontal pathogen adhesion compared to the titanium surface [21], alongside similar bone–implant contact compared to the titanium fixture with an almost overlapping range [22].

Additionally, Scarano et al. reported in a rabbit study that zirconia implants had about 68.4% bone–implant contact with evidence of contact osteogenesis without fibrous tissue interposition [23].

Zirconia material is distinguished by its clear ivory appearance, which is very similar to the natural color of the teeth and is characterized by an intrinsic strength and physical resistance to the loading [24,25,26]; as a result, it has been introduced as a restorative material for dental crowns, bars, abutments, and specially designed drills and burs [26,27,28,29,30,31,32,33,34]. Therefore, zirconia has recently gained further attention in the scientific community by growing research activities to confirm the clinical effectiveness of zirconia as a dental implant material.

Although citations are not an infallible metric to determine whether research is beneficial to researchers and clinicians, citations and citation analysis can quantify an article’s influence, author, subject of debate, country, journal, or a specialty [35,36]. Based on citation analysis, the bibliometric analysis aims to provide information about the trend in a research field and demonstrates its growth and development [37]; the number of citations received, researcher H-index, and journal impact factor are the most common bibliometric evaluation variables and considered as a scientific productivity score for the scientometric evaluation [38].

With the significant increase in the published articles on dental implants, recognizing trends and advances in a research field is critical and relevant to the needs of dental practitioners and researchers [39,40]. In this sense, bibliometric analysis is a useful tool for this purpose [41,42].

As far as we know, the trends and advances in zirconia dental implants have not been studied before; hence this study aimed to evaluate the bibliometric output of clinical research and co-authors in the field of zirconia dental implant rehabilitations.

## 2. Materials and Methods

We reported this bibliometric study in compliance with the Standards for Reporting Qualitative Research (SRQR) [43] and the Preferred Reporting Items for Systematic Reviews and Meta-Analyses (PRISMA) guidelines [44].

### 2.1. Search Strategy

An online literature search was conducted in Elsevier’s Scopus and Clarivate Analytics’ Web of Science (WoS) until 5 October 2020. We used the medical terms (MeSH) feature in the Cochrane Library to obtain the available synonyms for our search terms to create a detailed search strategy (Table 1).

### 2.2. Data Extraction and Bibliometric Parameters

We used a specially built Excel file (Microsoft, Redmond, WA, USA) to collect the findings of the literature search. The file contained the following information: abstracts, year of publication, indexed keywords, journal name, citations as well as all co-author bibliometric data (H-index, number of papers related to zirconia implant, the total number of papers, citation of paper regarding zirconia implant, and citation of paper regarding zirconia implant). Authors with the highest quantity of clinical studies regarding zirconia dental implants were evaluated and measured the average, the standard deviation, minimum and maximum of topic paper, total papers, topic citations, overall citations, and H-index. Moreover, we evaluated the scientific trend of the included study according to the year of publication and journal details (full title, the impact factor (IF), and rank) based on the Clarivate Analytics report for 2019 with selected categories: “Dentistry, Oral Surgery & Medicine”, study design, number of citations received, marginal bone loss, survival rate, failure, and study follow-up.

### 2.3. Study Selection

We screened the literature search results in two steps, where the first phase was the screening of the title and abstract by paired reviewers separately. Then, the second phase was a full-text assessment by two expert reviewers (L.F and A.S). The reference list of the studies included in the full-text screening was hand-screened for potential additional studies. In this bibliometric study, inclusion criteria were only clinical studies (either prospective or retrospective) without time restrictions. Exclusion criteria were animal studies, in vitro studies, literature reviews, systematic reviews, short communications, personal opinion, letters, book chapters, and non-English studies.

### 2.4. Data Analysis

We used VOSviewer software (version 1.6.8; Leiden University, Leiden, The Netherlands) to visualize a term map analyzing keywords from the data obtained. “Create Map” function was used to analyze the data by using the “Citation” type and setting the unit of analysis as a “number of citations.” In the keyword map, the node’s size reflects the number of received citations, as the larger size indicates the author with the highest citations. Furthermore, keywords that often appeared together were classified as the same color in network visualization mode [45,46].

## 3. Results

### 3.1. Study Selection

**Figure 1:**
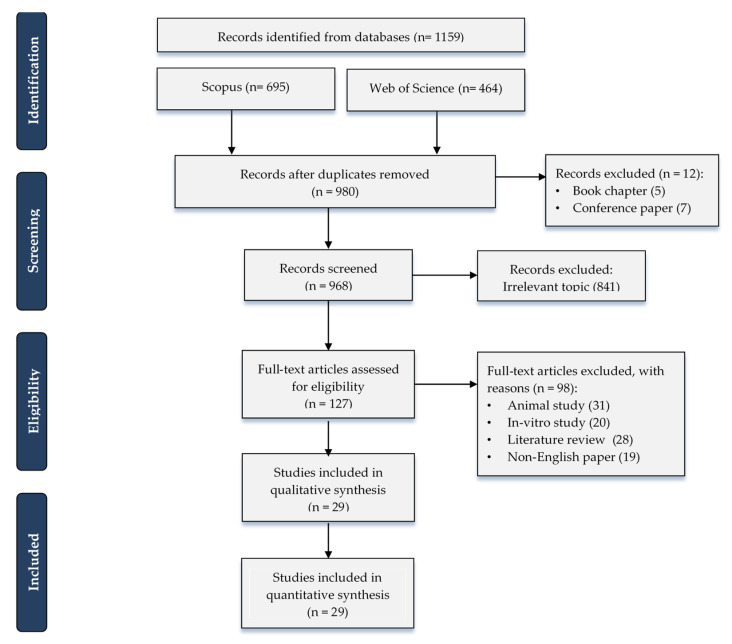
Preferred Reporting Items for Systematic Reviews and Meta-Analyses (PRISMA) flow chart demonstrates the process of literature search and study selection.

A total of 1159 references were collected from electronic databases in which (n = 185) were omitted due to duplication. By title and abstract, 968 articles were screened and 841 excluded as irrelevant topics. By the full-text screening of 127 papers, 29 studies were included in this bibliometric study [47,48,49,50,51,52,53,54,55,56,57,58,59,60,61,62,63,64,65,66,67,68,69,70,71,72,73,74,75] excluding the remaining 98 articles because they did not meet our inclusion criteria (Figure 1).

### 3.2. Study Characteristics

The included studies showed wide variability in the study design, presence/absence of a control group, experimental site, type of prosthetic rehabilitation, prosthetic connection (one-piece or two-piece), follow-up period, and different methods for evaluating the effectiveness of research. Although these differences exist, most studies reported favorable outcomes for the use of zirconia implants in oral rehabilitation. The main characteristics of the included studies are summarized in Table 2.

A total of 21 studies evaluated monolithic or one-piece zirconia implants [47,48,49,51,52,53,54,56,57,58,60,62,64,66,70,71,72,73,74,76], two of which had titanium implants as their control and showed no significant difference in survival rate and marginal bone loss between groups (*p* > 0.05) [64,70]. Two studies evaluated the immediate loading of zirconia implants [53,64]: one study compared it to the non-occlusal loading procedure [64], while the other study compared it with the standard loading protocol [57]. Furthermore, 26 papers assessed the cylindrical microgeometry of zirconia implants [47,48,49,50,51,52,53,54,55,56,57,58,59,60,61,62,63,64,65,67,70,71,72,73,74,75], while three studies evaluated the root-analog zirconia implants obtained by a three-dimensional scan [66,68,69]. However, Akça et al. and Pirker et al. reported the lowest marginal bone loss after two years (0.31 ± 0.24 and 0.5 ± 0.7 mm, respectively), in which Akça et al. used specially designed titanium–zirconia alloy implants [47], and Pirker et al. used specially designed root-analog zirconia implants with a micro-retention surface in a fresh extraction socket [69].

### 3.3. Growth of Publications

**Figure 2:**
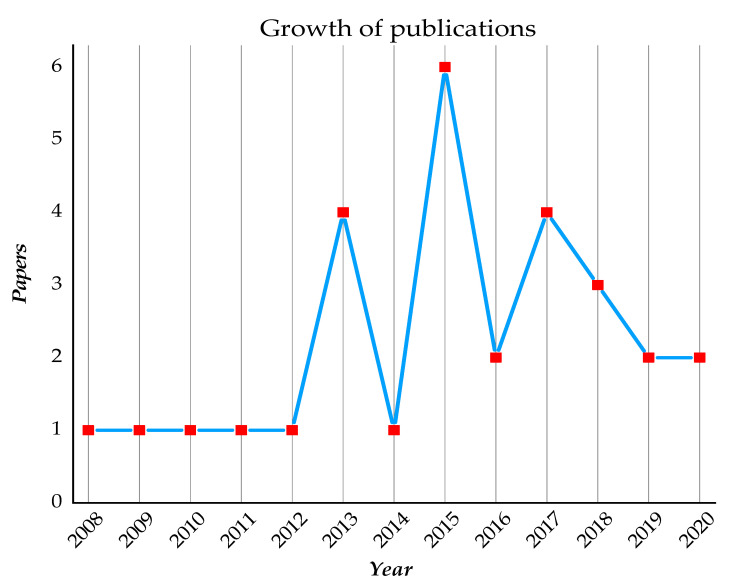
Publication trend of the clinical studies on the zirconia implants.

In total, 29 clinical studies were published between 2008 and 2020, in which 19 papers (65.5%) were published in the last five years and ten papers published before 2015. The highest number of published studies was in 2015 (*n* = 6, 20.6%) followed by 2013 and 2017 (*n* = 4, 13.7% for each) (Figure 2).

### 3.4. Journal of Publication

**Figure 3:**
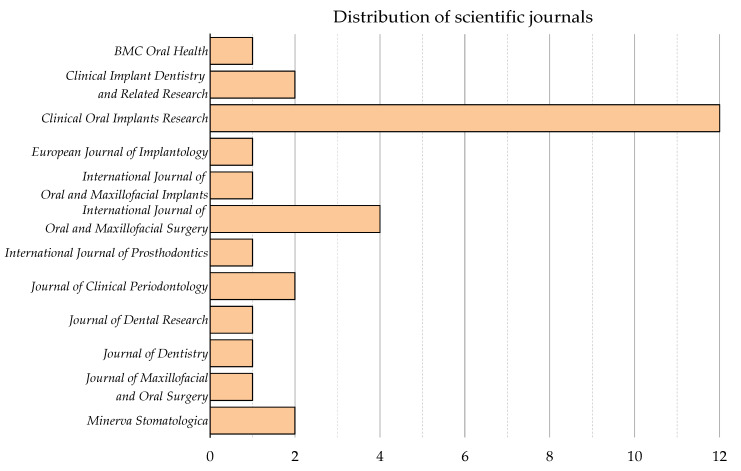
Contribution journals in clinical research on zirconia implants.

The clinical studies on the use of zirconia dental implants for oral rehabilitation were published across ten peer-reviewed journals. The journal with the largest number of publications was “Clinical Oral Implants Research” (*n* = 12, 41%), followed by “International Journal of Oral and Maxillofacial Surgery” (*n* = 4, 13.7%) (Figure 3).

The majority of publications were published in Q1 journals (*n* = 25, 86%), while the journal with the highest impact factor was “Journal of Clinical Periodontology” (IF = 5.241), which had two articles.

### 3.5. Study Design and Level of Evidence

All included studies were prospective, while the most common study design of clinical research on zirconia implants was cohort study (*n* = 12, 41.4%), followed by case series (*n* = 9, 31%), and RCT (*n* = 5, 17%). According to the hierarchy of evidence levels (Is) [77,78], the available evidence supporting the use of zirconia implants is 17% level II, 41.4% EL IV, and the remaining EL VI.

### 3.6. Contribution of Countries and Institutions

The majority of the studies originated from institutions in Germany (*n* = 14, 48.3%), followed by Switzerland, (*n* = 6, 20.7%), and Austria (*n* = 5, 17%), where the most productive institution was the Medical Center of University of Freiburg (*n* = 8, 27.6%), followed by the Center of Dental Medicine, University of Zürich (*n* = 5, 17%). While many of the included studies were funded, the most funding support for included research was provided by VITA Zahnfabrik—H. Rauter GmbH & Co. KG, Bad Säckingen, Germany (*n* = 5, 17%) (Table 3).

### 3.7. Bibliometric Assessment

A total of 29 articles with total citations_[Scopus]_ ranged from 0 to 176 (mean 57.28 ± 42.18), while the number of citations_[Scopus]_ received by each paper ranged from 0 to 69 (mean 21.3 ± 20). The top-cited study was the RCT of Cannizzaro et al. (2010) (*n*_[Scopus]_ = 69) [54], followed by the prospective case series of Payer et al. (2012) (*n*_[Scopus]_ = 61) [66], and Pirker et al. (2009) (*n*_[Scopus]_ = 58) [69].

**Figure 4:**
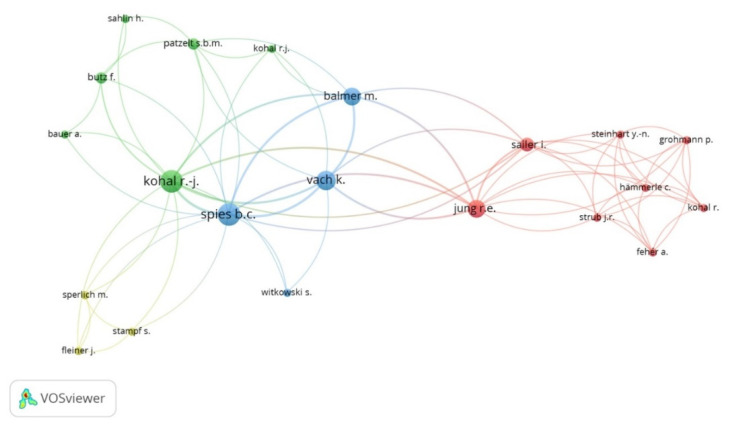
Network analysis of the authors with the largest number of clinical studies on zirconia implants.

**Figure 5:**
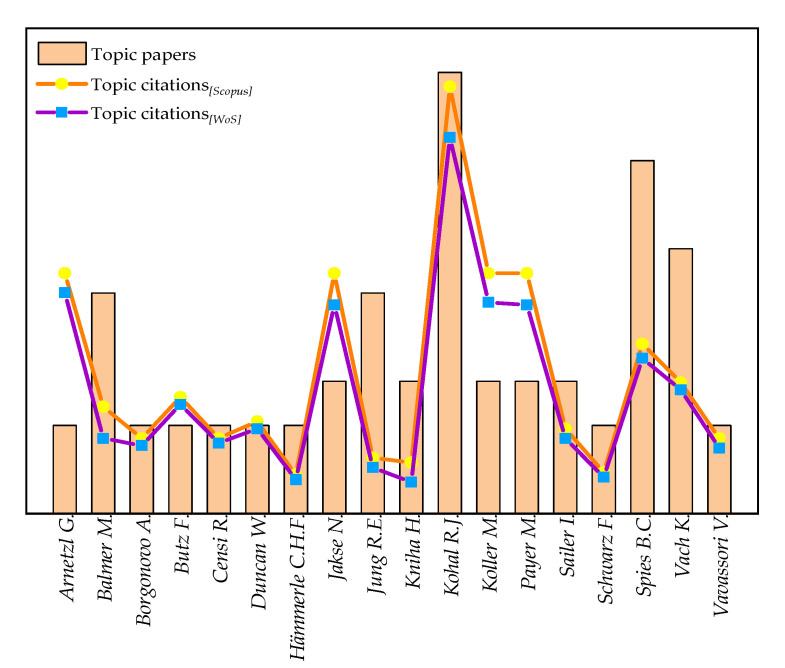
Bibliometric variables for authors with the highest number of topic papers.

However, the author with the highest number of clinical research on zirconia implants was Kohal R.J. (*n* = 10), followed by Spies B.C. (*n* = 8) and Vach K. (*n* = 6), while the top-cited author of clinical studies on zirconia implants was Kohal R.J. (*n*_[WoS]_ = 155), followed by Arnetzl G. (*n*_[WoS]_ = 91), Koller M. (*n*_[WoS]_ = 87), Payer M., and Jakse N. (*n*_[WoS]_ = 86 for each) (Figure 4 and Figure 5).

**Figure 6:**
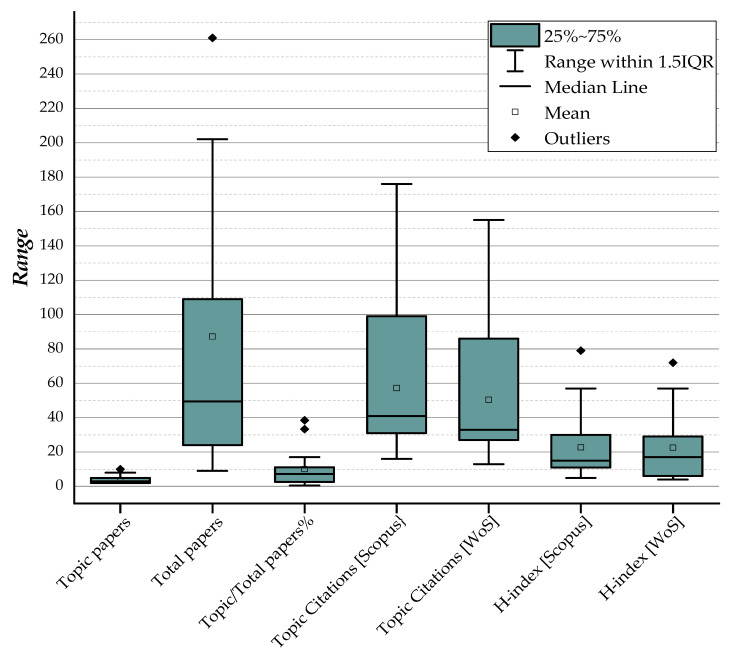
Box plots summarize the bibliometric variables of the authors with the largest number of studies.

The authors’ H-index_Scopus_ ranged from one to 79 (mean 22.67 ± 19.96), and the author with the most bibliometric characteristics was Hämmerle C.H.F., who had 364 publications (two of which were clinical studies on zirconia implants) with 8311 total citations and H-index_Scopus_ = 79 (Table 4 and Figure 6).

## 4. Discussion

The present study carried out a bibliometric evaluation of clinical research on zirconia implant rehabilitation, highlighting the significant heterogeneity of the included studies, which revealed considerable variations in methodology, technical approaches, follow-up, and control group involvement. Our findings indicate that there is a trend for zirconia implants in oral rehabilitation as there has been an increase in about 180% of the studies published in the last five years.

The included studies reported a survival rate for zirconia implants ranging from 87% to 100% with follow-up periods from one to 7.8 years, while the least survival rate reported in RCT by Siddiqi et al. was 67.6% after one-year follow-up (i.e., 16 zirconia implants failed out of 68) [70]. This RCT aimed to study the effectiveness of zirconia vs. titanium implants restored with one-piece ball-abutment in mandibular and maxillary overdentures, while this high decrease in the survival rate was for both groups (i.e., 67.6% for zirconia implants and 66.7% for titanium implants); the outcomes of maxillary rehabilitation were worse than the mandible, while no mechanical fractures of the fixtures were reported [70].

Although one-piece and two-piece zirconia implants have been evaluated, the lower marginal bone loss and higher survival rates were observed in studies of one-piece zirconia implant rehabilitation on a single tooth or three element prosthetic rehabilitation [59,61]. However, the studies did not report any differences in the marginal bone loss and survival rate between the single crown and the fixed multiple zirconia implant recovery, while the prosthetic connection appears to have no apparent effect on these parameters [48]. Additionally, Lorenz et al. showed no significant difference in marginal bone loss with a total of 83 zirconia implants compared to natural teeth after 7.8 years of function [47], and the marginal bone loss was similar in the other studies, which was less than 1 mm in the first year and stabilized in subsequent functional loading [47,48,49,52,56,57,58,64,69,73,74]. Moreover, the prospective study by Kniha et al. contained the largest sample size of the included studies involving 81 patients with 105 implants for fixed rehabilitation, who reported a significant decrease of 0.66 ± 0.30 mm with a survival rate of 100% after three years [58].

However, the most common complication (70%) was the failure of implant osseointegration as 17 studies reported a loss of at least one implant in the first six months [48,49,50,51,53,54,55,56,59,60,61,63,64,66,67,69,70,72,73,74].

As previously reported for titanium dental implant threads, microgeometry appears to have a significant effect on the osseointegration of zirconia implants [79,80], whereas a more retentive surface resulted in an increased survival rate compared to a sandblasted surface only [68,69].

Although all clinical research included in this analysis was screened and selected from the Scopus and Web of Science databases, which may avoid restriction in each database [39,81], our investigation has further limitations. First, the year of publication, which is a reliable indicator of the number of citations received, as older papers receive more citations than recent publications because there is more time to cite them, regardless of their impact [82,83]. Second, open access policies have a significant influence on the citations received in the evaluated papers [84,85,86], as a result, we found large heterogeneity in Topic/Total Citations% and co-authors’ H-index.

## 5. Conclusions

This was the first study highlighting bibliometric output of clinical research and co-authors in the field of zirconia dental implants and shows a strong interest in the development of research into the clinical application of zirconia dental implants, as evidenced by the increase in the number of scientific papers published in the last ten years.

## Figures and Tables

**Table 1:** Search strategy used for each database.

**Scopus**	TITLE-ABS-KEY (“Zirconia” OR “Zirconium” OR “Zircon*”) AND TITLE-ABS-KEY (“Dental implant” OR “Dental implants” OR “Oral implant” OR “Oral implants” OR “Implant dentistry” OR “Dental implantology” OR “Dental Implantation” OR “Osseointegrated” OR “Osseointegrated Dental Implantation”) AND TITLE-ABS-KEY (“Intervention Study” OR “Clinical Trial” OR “Controlled Clinical Trial” OR “Randomized Controlled Trials OR “Non-Randomized Clinical Trial” OR “Nonrandomized Clinical Trial” OR “Quasi-Experimental” OR “Observational Study” OR “Prospective Study” OR “Prospective” OR “Retrospective Study” OR “Retrospective” OR “Comparative Study” OR “Multicenter Studies” OR “Epidemiologic Study” OR “Epidemiological Studies” OR “Cohort Study” OR “Case Studies” OR “Follow-Up Study” OR “Case-Control Study” OR “Case Report” OR “Case Series” OR “Pilot Study”)
**Web of Science**	TS = (“Zirconia” OR “Zirconium” OR “Zircon*”) AND TS = (“Dental implant” OR “Dental implants” OR “Oral implant” OR “Oral implants” OR “Implant dentistry” OR “Dental implantology” OR “Dental Implantation” OR “Osseointegrated” OR “Osseointegrated Dental Implantation”) AND TS = (“Intervention Study” OR “Clinical Trial” OR “Controlled Clinical Trial OR “Randomized Controlled Trials” OR “Non-Randomized Clinical Trial” OR “Nonrandomized Clinical Trial” OR “Quasi-Experimental” OR “Observational Study” OR “Prospective Study” OR “Prospective” OR “Retrospective Study” OR “Retrospective” OR “Comparative Study” OR “Multicenter Studies” OR “Epidemiologic Study” OR “Epidemiological Studies” OR “Cohort Study” OR “Case Studies” OR “Follow-Up Study” OR “Case-Control Study” OR “Case Report” OR “Case Series” OR “Pilot Study”) **Timespan:** All years. **Databases:** WOS, ARCI, BCI, KJD, MEDLINE, RSCI, SCIELO, ZOOREC. Search language = Auto.

**Table 2:** Main characteristics of the clinical research included (Zir: Zirconia implant group, Tit: Titanium implant group, IF: impact factor, RCT: Randomized controlled trial).

Authors (Year) [Ref]	Journal			Cited By	Study Design	Patients (Implants)	Test	Control	Marginal Bone Loss (Mean ± SD)	Survival Rate	Failure	Follow Up
	Full Title	Rank	IF									
Pirker et al. (2008) [68]	International Journal of Oral and Maxillofacial Surgery	33	2.068	50	Case report	1 (1 Implant)	Microretention and sandblasted root-analogue zirconia implant	-	-	100%	-	2 years
Pirker et al. (2009) [69]	International Journal of Oral and Maxillofacial Surgery	33	2.068	58	Prospective Case Series	18 (18 Implants)	Microretention and sandblasted root-analogue zirconia implants	Sandblasted root-analogue zirconia implants	0.5 ± 0.7 mm	Test: 92% Control: 0%	Test: 1 implant Control: All implants (6)	2 years
Cannizzaro et al. (2010) [54]	European Journal of Implantology	-	-	69	Multicenter RCT	40 (40 Implants)	Immediate occlusal loading zirconia Implants	Immediate non-occlusal loading zirconia Implants	Test: 0.90 ± 0.48 mm Control: 0.72 ± 0.59 mm	88.50%	5 implants (12.5%): Test: 3 Implants Control: 2 Implants	1 year
Borgonovo et al. (2011) [51]	Minerva Stomatologica	-	-	21	Prospective Case Series	16 (26 Implants)	One-piece yttrium stabilized zirconia implants	-	-	96.16%	1 Implant osseointegration failure	2 years
Payer et al. (2012) [66]	Clinical Oral Implants Research	8	3.723	61	Prospective Case Series	20 (20 Implants)	One-piece zirconia implants	-	1.29 ± 0.73 mm	95%	1 Implant osseointegration failure	2 years
Akça et al. (2013) [47]	International Journal of Oral and Maxillofacial Implants	24	2.32	8	Prospective Case Series	23 (52 Implants)	-	-	0.32 ± 0.24 mm	100%	No failure	2 years
Borgonovo et al. (2013) [52]	Minerva Stomatologica	-	-	10	Prospective Case Series	6 (14 Implants)	One-piece yttrium stabilized zirconia implants	-	0.67 ± 0.51 mm	100%	No failure	4 years
Kohal et al. (2013) [59]	Journal of Clinical Periodontology	2	5.241	47	Prospective Case Series	28 (56 Implants)	One-piece yttria-stabilized tetragonal zirconia implants	-	1.95 ± 0.65	98.20%	1 Implant osseointegration failure	1 year
Osman et al. (2013) [63]	International Journal of Prosthodontics	61	1.49	6	Pilot study	4 (28 Implants)	One-piece zirconia implants for ball abutment	-	-	85.70%	4 Implants	1 year
Osman et al. (2014) [64]	Clinical Oral Implants Research	8	3.723	34	RCT	19 (129 Implants)	One-piece zirconia implants for ball-abutment	One-piece titanium implants for ball-abutment	Zir: 0.42 ± 0.40 Tit: 0.18 ± 0.47	Zir: 90.9%Tit: 95.8%	Zir: 21Implants (3 fractured) Tit: 10Implants	1 year
Becker et al. (2015) [50]	Clinical Oral Implants Research	8	3.723	15	Prospective Cohort Study	52 (52 Implants)	Two-piece zirconia implants	-	-	95.80%	2 Implants	2 years
Cionca et al. (2015) [55]	Clinical Oral Implants Research	8	3.723	43	Prospective Case Series	32 (49 Implants)	Two-piece zirconia implants	-	-	87%	6 Implants	1 year
Jung et al. (2015) [56]	Clinical Oral Implants Research	8	3.723	27	Prospective Cohort Study	60 (71 Implants)	Immediate one-piece zirconia implants	-	0.78 ± 0.79 mm	98.30%	1 implant osseointegration failure	1 year
Payer et al. (2015) [67]	Clinical Oral Implants Research	8	3.723	41	RCT	22 (31 Implants)	Two-piece zirconia implants	Two-piece titanium implants	Zir: 1.48 ± 1.05 Tit: 1.43 ± 0.67	Zir: 93.3% Tit: 100%	Zir: 1 Implant Tit: No failure	2 years
Siddiqi et al. (2015) [70]	Clinical Implant Dentistry and Related Research	9	3.396	17	RCT	22 (150 Implants)	One-piece zirconia implants for ball-abutment	Titanium implants for one-piece ball-abutment	Zir: 2.23 ± 0.69 Tit: 1.59 ± 0.33	Zir: 67.6% Tit: 66.7%	Zir: 16 Implants Tit: 7 Implants	1 year
Spies et al. (2015) [73]	Journal of Dental Research	3	4.914	22	Prospective Cohort Study	40 (53 Implants)	One-piece alumina-toughened zirconia implant	-	0.79 ± 0.47 mm	94.2%	3 Implants osseointegration failure	3 years
Patankar et al. (2016) [65]	Journal of Maxillofacial and Oral Surgery	-	-	3	Case report	1 (1 Implant)	Microretention and sandblasted root-analogue zirconia implant	-	-	100%	-	1.5 year
Spies et al. (2016) [74]	Clinical Oral Implants Research	8	3.723	13	Prospective Cohort Study	27 (27 Implants)	Immediate one-piece alumina-toughened zirconia implant	-	0.77 ± 0.31 mm	88.90%	3 Implants osseointegration failure	1 year
Kniha et al. (2017) [58]	International Journal of Oral and Maxillofacial Surgery	33	2.068	9	Prospective Cohort Study	81 (105 Implants)	Zirconia implants	-	0.66 ± 0.33 mm	100%	No failure	3 years
Kniha et al. (2017) [57]	International Journal of Oral and Maxillofacial Surgery	33	2.068	9	Prospective Cohort Study	78 (82 Implants)	Immediate loading one-piece zirconia implants	Delayed one-piece zirconia implants	Immediate: 0.76 ± 1.13 mm Delayed: 0.83 ± 0.65 mm	Immediate: 100% Delayed: 100%	No failure	1 year
Spies et al. (2017) [71]	Journal of Dentistry	10	3.242	6	Prospective Case Series	60 (71 Implants)	One-piece zirconia oral implants	-	-	100%	No failure	3 years
Spies et al. (2017) [75]	Clinical Oral Implants Research	8	3.723	11	Prospective Case Series	13 (26 Implants)	One-piece zirconia implants	-	-	100%	No failure	5 years
Balmer et al. (2018) [49]	Clinical Oral Implants Research	8	3.723	11	Prospective Multicenter Cohort Study	60 (71 Implants)	One-piece immediate loading zirconia implants	-	0.70 ± 0.72 mm	98.50%	1 Implant osseointegration failure	3 years
Bormann et al. (2018) [53]	BMC Oral Health	38	1.911	7	Prospective Multicenter Cohort Study	44 (44 Implants)	Zirconia implants	-	0.97 ± 0.88 mm	97.50%	1 Implant	3 years
Kohal et al. (2018) [60]	Journal of Clinical Periodontology	2	5.241	5	Prospective Cohort Study	65 (65 Implants)	Immediate loading one-piece zirconia implants	-	1.45 ± 1.96 mm	90.80%	6 Implants	3 years
Lorenz et al. (2019) [62]	Clinical Implant Dentistry and Related Research	9	3.396	4	Prospective Cohort Study	28 (83 Implants)	Zirconia implants	Natural teeth	1.2 ± 0.76 mm	100%	No failure one peri-implantitis resistant to therapies	7.8 years
Spies et al. (2019) [72]	Clinical Oral Implants Research	8	3.723	5	Prospective Multicenter Cohort Study	45 (45 Implants)	Zirconia implants	-	-	97.5 ± 2.47%.	Chipping (n = 19) occlusal roughness (n = 35)	5 years
Balmer et al. (2020) [48]	Clinical Oral Implants Research	8	3.723	4	Prospective Multicenter Cohort Study	60 (71 Implants)	Single crown one-piece zirconia implant	Multiple prostheses one-piece zirconia implant	0.7 ± 0.6 mm	98.4%	1 Implant	5 years
Koller et al. (2020) [61]	Clinical Oral Implants Research	8	3.723	0	Pilot RCT	22 (31 Implants)	Two-piece zirconia implants	Two-piece titanium implants	Zir: 1.38 ± 0.81 Tit: 1.17 ± 0.73 mm	Zir: 87.5%Tit: 93.3%	Zir: 2 Implants Tit: 1 Implant	6.67 years

**Table 3:** Contribution of countries and institutions to clinical studies on zirconia implants.

Country	Institution	Study [Ref]	Funding
Germany	Universitätsklinikum Düsseldorf, Düsseldorf	Becker et al., 2015 [50]	ZV3 Zircon Vision GmbH, Wolfratshausen, Germany
	University Hospital Aachen, Aachen	Kniha et al., 2017 [57]	No Funding
	Friedrich-Alexander-University Erlangen-Nürnberg	Kniha et al., 2017 [58]	
	Johann-Wolfgang Goethe University, Frankfurt/Main	Lorenz et al., 2019 [62]	
	Hannover Medical School, Hannover	Bormann et al., 2018 [53]	Institut Straumann AG, Basel, Switzerland
	School of Dentistry, Albert-Ludwigs University, Freiburg	Kohal et al., 2013 [59]	Nobel Biocare AB, Göteborg, Sweden
	Medical Center of University of Freiburg, Freiburg	Kohal et al., 2018 [60]	
		Spies et al., 2015 [73]	Metoxit AG (Thayngen, Switzerland)
		Spies et al., 2016 [74]	
		Spies et al., 2017 [75]	Ivoclar Vivadent
Germany and Switzerland	Medical Center of University of Freiburg, Freiburg and Center of Dental Medicine, University of Zürich, Zürich	Spies et al., 2017 [71]	VITA Zahnfabrik—H. Rauter GmbH & Co. KG, Bad Säckingen, Germany
		Balmer et al., 2018 [49]	
		Spies et al., 2019 [72]	
		Balmer et al., 2020 [48]	
Switzerland	School of Dental Medicine, University of Geneva, Geneva	Cionca et al., 2015 [55]	Dentalpoint AG, Zürich, Switzerland
	Center of Dental Medicine, University of Zürich, Zürich	Jung et al., 2015 [56]	VITA Zahnfabrik—H. Rauter GmbH & Co. KG, Bad Säckingen, Germany
Austria	Alfred Kocher, Medical University Vienna, Vienna	Pirker et al., 2008 [68]	No Funding
		Pirker et al., 2009 [69]	
	School of Dentistry, Medical University Graz, Graz	Payer et al., 2012 [66]	Bredent medical GmbH, Senden, Germany
		Payer et al., 2015 [67]	Ziterion GmbH, Uffenheim, Germany
		Koller et al., 2020 [61]	
Italy	Private practice	Cannizzaro et al., 2010 [54]	Partially supported by Z-systems
	School of Dentistry, University of Milan, Milan	Borgonovo et al., 2011 [51]	Not reported
		Borgonovo et al., 2013 [52]	
New Zealand	Oral Implantology Research Group, Sir John Walsh Research Institute, School of Dentistry, University of Otago	Osman et al., 2013 [63]	Oral Implantology Research Group, Sir John Walsh Research Institute, School of Dentistry, University of Otago and Southern Implants
		Osman et al., 2014 [64]	
		Siddiqi et al., 2015 [70]	
India	BV Dental College and Hospital, Pune	Patankar et al., 2016 [65]	No Funding
Turkey	Faculty of Dentistry, Hacettepe University	Akça et al., 2013 [47]	No Funding

**Table 4:** General bibliometric variables for authors with the largest number of topic papers.

Author	Topic Papers	Total Papers	Topic/Total Papers %	Topic Citations [Scopus]	Topic Citations [WoS]	Total Citations [Scopus]	Total Citation [WoS]	Topic/Total Citations % [Scopus-WoS]	H-index [Scopus]	H-index [WoS]
Kohal R.J.	10	109	9.17%	176	155	3053	2975	[5.76–5.21%]	30	29
Spies B.C.	8	47	17.02%	70	64	452	448	[15.49–14.29%]	14	14
Vach K.	6	73	8.22%	54	51	661	820	[8.17–6.21%]	15	16
Balmer M.	5	13	38.46%	44	31	256	130	[17.19–23.84%]	7	5
Jung R.E.	5	202	2.48%	23	19	8359	9126	[0.28–0.21%]	47	57
Jakse N.	3	70	4.29%	99	86	1114	981	[8.89–8.77%]	18	18
Kniha H.	3	28	10.71%	21	13	664	726	[3.16–1.779%]	11	13
Koller M.	3	9	33.33%	99	87	166	312	[59.64–27.88%]	5	5
Payer M.	3	48	6.25%	99	86	770	1484	[12.86–5.79%]	15	23
Sailer I.	3	113	2.65%	35	31	6027	5713	[0.58–0.54%]	34	33
Arnetzl G.	2	40	5.00%	99	91	437	183	[22.65–49.72%]	11	6
Butz F.	2	24	8.33%	48	45	1248	1441	[3.85–3.12%]	18	19
Censi R.	2	18	11.11%	31	29	169	95	[18.34–30.52%]	8	4
Duncan W.	2	87	2.30%	38	35	2163	1356	[1.76–2.58%]	20	19
Hämmerle C.H.F.	2	364	0.55%	16	14	18,311	16,032	[0.09–0.08%]	79	72
Schwarz F.	2	261	0.77%	17	15	9093	9169	[0.19–0.16%]	57	57
Vavassori V.	2	12	16.67%	31	27	136	96	[22.79–28.12%]	7	5
Borgonovo A.	2	51	3.92%	31	28	535	449	[5.79–6.23%]	12	11
**Summary** **(Mean ± SD)**	3.61 ± 2.33	87.17 ± 96.37	10.07 ± 0.11%	57.28 ± 42.18	50.39 ± 37.68	2978.56 ± 4750.05	2863.11 ± 4386.90	-	22.67 ± 19.96	22.56 ± 20.14
